# I_1_ Imidazoline Receptor: Novel Potential Cytoprotective Target of TVP1022, the S-Enantiomer of Rasagiline

**DOI:** 10.1371/journal.pone.0047890

**Published:** 2012-11-15

**Authors:** Yaron D. Barac, Orit Bar-Am, Esti Liani, Tamar Amit, Luba Frolov, Elena Ovcharenko, Itzchak Angel, Moussa B. H. Youdim, Ofer Binah

**Affiliations:** 1 Department of Physiology and Biophysics, Faculty of Medicine, Technion, Haifa, Israel; 2 Eve Topf and National Parkinson Foundation Center For Neurodegenerative Diseases Research and Department of Pharmacology, Ruth & Bruce Rappaport Faculty of Medicine, Technion, Haifa, Israel; 3 A.P.C.T. Ltd., Ness-Ziona, Israel; Massachusetts Eye & Ear Infirmary, Harvard Medical School, United States of America

## Abstract

TVP1022, the S-enantiomer of rasagiline (Azilect®) (N-propargyl-1R-aminoindan), exerts cyto/cardio-protective effects in a variety of experimental cardiac and neuronal models. Previous studies have demonstrated that the protective activity of TVP1022 and other propargyl derivatives involve the activation of p42/44 mitogen-activated protein kinase (MAPK) signaling pathway. In the current study, we further investigated the molecular mechanism of action and signaling pathways of TVP1022 which may account for the cyto/cardio-protective efficacy of the drug. Using specific receptor binding and enzyme assays, we demonstrated that the imidazoline 1 and 2 binding sites (I_1_ & I_2_) are potential targets for TVP1022 (IC_50_ = 9.5E-08 M and IC_50_ = 1.4E-07 M, respectively). Western blotting analysis showed that TVP1022 (1–20 µM) dose-dependently increased the immunoreactivity of phosphorylated p42 and p44 MAPK in rat pheochromocytoma PC12 cells and in neonatal rat ventricular myocytes (NRVM). This effect of TVP1022 was significantly attenuated by efaroxan, a selective I_1_ imidazoline receptor antagonist. In addition, the cytoprotective effect of TVP1022 demonstrated in NRVM against serum deprivation-induced toxicity was markedly inhibited by efaroxan, thus suggesting the importance of I_1_imidazoline receptor in mediating the cardioprotective activity of the drug. Our findings suggest that the I_1_imidazoline receptor represents a novel site of action for the cyto/cardio-protective efficacy of TVP1022.

## Introduction

Much of the morbidity and mortality resulting from cardiovascular diseases is attributable to acute ischemic events leading to myocardial infarction and death of cardiac myocytes [1], [2]. The current gold standard treatment is enabling reperfusion by using either per-cutaneous tools (e.g., stents) or by means of coronary artery bypass graft surgery. Although reperfusion is a necessity, the accompanying ischemia and reperfusion injury is often devastating. Therefore, protecting the heart from I/R injury has been the focus of intense research over the past years. However, despite numerous publications and many successful preclinical experiments, thus far no effective cardioprotective drug has found its way to the clinical practice [3], [4]. We have reported previously that the compound TVP1022, which is the S-enantiomer of rasagiline (Azilect®) (N-propargyl-1R-aminoindan; a novel FDA-approved anti-Parkinsonian drug) possesses cytoprotective efficacy in a variety of cardiac and neuronal experimental models [5], [6], [7], [8], [9]. We have demonstrated that although TVP1022 is ∼1000 times less potent than rasagiline as a monoamine oxidase-B inhibitor [9], [10], it exerts a prominent effective neuroprotective and anti-apoptotic activities in neuronal cell cultures in response to various neurotoxins, and in *in vivo* model of head injury [5], [8], [9]. Studies on structure-activity relationship revealed that the neuroprotective effect of TVP1022 is associated mainly with its propargyl moiety, and is ascribed, at least partly, to the stabilization of mitochondrial membrane potential, induction of Bcl-2 and activation of p42/44 mitogen-activated protein kinase (MAPK) and protein kinase C (PKC) signaling pathways [7], [9], [10], [11].

In agreement with its cytoprotective efficacy, TVP1022 was further found to exert cardioprotective effects against doxorubicin (ananthracycline chemotherapeutic agent), and serum deprivation-induced apoptosis in cultured neonatal rat ventricular myocytes (NRVM) [12]. It was demonstrated that pretreatment of NRVM cultures with TVP1022 or propargylamine inhibited the increase in cleaved caspase 3 levels and prevented the decline in Bcl-2/Bax ratio [12]. In addition, in both H9c2 cardiomyoblasts and NRVM, TVP1022 attenuated serum deprivation- and H_2_O_2_ -induced apoptosis. Specifically, TVP1022 preserved mitochondrial membrane potential and Bcl-2 levels, inhibited mitochondrial cytochrome c release and the increase in cleaved caspase 9 and 3 levels and enhanced the phosphorylation of PKC and glycogen synthase kinase-3β [13]. TVP1022 was also found to attenuate the functional derangements (e.g., intracellular Ca^2+^transients and contractions properties and intercellular coupling) caused by doxorubicin in NRVM [14]. Our recent *in vivo* study showed that in a rat model of I/R, TVP1022 provided prominent cardioprotection, evidenced by a reduction in the infarct size, attenuation of the decline in ventricular function and diminution of mitochondrial damage caused by I/R, thus rendering this molecule a potentially novel cardioprotective drug [13].

**Table 1:** Experimental conditions of binding assays.

Assay	Ligand	Conc.(nM)	Non specific/Conc. (µM)	Inc.(min/°C)	Origin
A_1_ *(h)*	[^3^H]DPCPX	1	DPCPX/1	60/22	CHO cells
A_2A_ *(h)*	[^3^H]CGS 21680	6	NECA/10	120/22	HEK293 cells
A_3_ *(h)*	[^125^I]AB-MECA	0.15	IB-MECA/1	120/22	HEK293 cells
α_1_ (non-selective)	[^3^H]prazosin	0.25	Prazosin/0.5	60/22	rat cerebral cortex
α_2_ (non-selective)	[^3^H]RX 821002	0.5	(–)epinephrine/100	60/22	rat cerebral cortex
β_1_ *(h)*	[^3^H](–)CGP 12177	0.15	alprenolol/50	60/22	HEK293 cells
β_2_ *(h)*	[^3^H](–)CGP 12177	0.2	alprenolol/50	120/22	CHO cells
AT_1_ *(h)*	[^125^I][Sar^1^,Ile^8^]-AT-II	0.05	angiotensin-II/10	120/37	HEK293 cells
AT_2_ *(h)*	[^125^I]CGP 42112A	0.04	angiotensin-II/1	180/37	CHO cells
BZD (central)	[^3^H]flunitrazepam	0.4	diazepam/3	60/4	rat cerebral cortex
B_1_ *(h)*	[^3^H]desArg^10^-KD	0.35	desArg^9^[Leu^8^]-BK/10	60/22	CHO cells
B_2_ *(h)*	[^3^H]bradykinin	0.2	Bradykinin/1	60/22	CHO cells
CB_1_ *(h)*	[^3^H]CP 55940	0.5	WIN 55212-2/10	120/37	CHO cells
CB_2_ *(h)*	[^3^H]WIN 55212-2	0.8	WIN 55212-2/5	120/37	CHO cells
CCK_A_ *(h)* (CCK_1_)	[^125^I]CCK-8s	0.08	CCK-8S/1	60/22	CHO cells
CCK_B_ *(h)* (CCK_2_)	[^125^I]CCK-8s	0.08	CCK-8S/1	60/22	CHO cells
CRF_1_ *(h)*	[^125^I]sauvagine	0.075	sauvagine/0.5	120/22	CHO cells
D_1_ *(h)*	[^3^H]SCH 23390	0.3	SCH 23390/1	60/22	CHO cells
D_2S_ *(h)*	[^3^H]spiperone	0.3	(+)butaclamol/10	60/22	HEK293 cells
D_3_ *(h)*	[^3^H]spiperone	0.3	(+)butaclamol/10	60/22	CHO cells
D_4.4_ *(h)*	[^3^H]spiperone	0.3	(+)butaclamol/10	60/22	CHO cells
ET_A_ *(h)*	[^125^I]endothelin-1	0.03	endothelin-1/0.1	120/37	CHO cells
ET_B_ *(h)*	[^125^I]endothelin-1	0.03	endothelin-1/0.1	120/37	CHO cells
GABA (non-selective)	[^3^H]GABA	10	GABA/100	60/22	rat cerebral cortex
AMPA	[^3^H]AMPA	8	L-glutamate/1	60/4	rat cerebral cortex
Kainate	[^3^H]kainic acid	5	L-glutamate/1	60/4	rat cerebral cortex
NMDA	[^3^H]CGP 39653	5	L-glutamate/100	60/4	rat cerebral cortex
H_1_ *(h)*	[^3^H]pyrilamine	3	pyrilamine/1	60/22	HEK293 cells
H_2_ *(h)*	[^125^I]APT	0.075	tiotidine/100	120/22	CHO cells
H_3_ *(h)*	[^3^H]N^α^-Me-histamine	1	(R)α-Me-histamine/1	60/22	CHO cells
I_1_	[^3^H]clonidine	15	rilmenidine/10	60/22	bovine adrenal glands
I_2_	[^3^H]idazoxan	2	cirazoline/10	30/22	rat cerebral cortex
LTB_4_ *(h)* (BLT_1_)	[^3^H]LTB_4_	0.2	LTB_4_/0.2	60/22	CHO cells
LTD_4_ *(h)* (CysLT_1_)	[^3^H]LTD_4_	0.3	LTD_4_/1	60/22	CHO cells
MC_4_ *(h)*	[^125^I]NDP-α-MSH	0.05	NDP-α-MSH/1	120/37	CHO cells
M (non-selective)	[^3^H]QNB	0.05	atropine/1	120/22	rat cerebral cortex
NK_1_ *(h)*	[^125^I]BH-SP	0.15	[Sar^9^,Met(O_2_)^11^]-SP/1	60/22	U-373MG cells
NK_2_ *(h)*	[^125^I]NKA	0.1	[Nle^10^]-NKA/10	60/22	CHO cells
NK_3_ *(h)*	[^3^H]SR 142801	0.4	SB 22200/10	120/22	CHO cells
Y (non-selective)	[^3^H]NPY	0.5	NPY/1	90/22	rat cerebral cortex
N(neuronal) (α4β2)	[^3^H]cytosine	1.5	nicotine/10	75/4	rat cerebral cortex
Opioid (non-selective)	[^3^H]naloxone	1	naloxone/1	40/22	rat cerebral cortex
ORL1 *(h)* (NOP)	[^3^H]nociceptin	0.2	nociceptin/1	60/22	HEK293 cells
PPARγ*(h)*	[^3^H]rosiglitazone	10	rosiglitazone/10	120/4	*E*.coli
PCP	[^3^H]TCP	5	MK 801/10	60/22	rat cerebral cortex
EP_4_ *(h)*	[^3^H]PGE_2_	1	PGE_2_/10	120/22	CHO cells
IP *(h)* (PGI_2_)	[^3^H]iloprost	10	iloprost/10	60/22	human platelets
P2X	[^3^H]α,β-MeATP	3	α,β-MeATP/10	120/4	rat urinary bladder
P2Y	[35S]dATPαS	10	dATPαS/10	60/22	rat cerebral cortex
5-HT (non-selective)	[^3^H]serotonin	2	serotonin**/**10	60/37	rat cerebral cortex
σ (non-selective)	[^3^H]DTG	8	haloperidol/10	120/22	rat cerebral cortex
Glucocorticoid *(h)*(GR)	[^3^H]]dexamethasone	1.5	triamcinolone/10	6/4	IM-9 cells (cytosol)
Estrogen *(h)* (ER)	[^3^H]estradiol	1	17-β-estradiol/6	20/4	MCF-7 cells (cytosol)
Progesterone *(h)* (PR)	[^3^H]R 5020	2	R 5020/1	20/4	MCF-7 cells (cytosol)
Androgen *(h)* (AR)	[^3^H]methyltrienolone	0.5	mibolerone/1	24/4	LNCaP cells (cytosol)
TRH_1_ *(h)*	[^3^H]Me-TRH	2	TRH/10	120/4	CHO cells
V_1a_ *(h)*	[^3^H]AVP	0.3	AVP/1	60/22	CHO cells
V_2_ *(h)*	[^3^H]AVP	0.3	AVP/1	120/22	CHO cells
Ca^2+^channel (L, DHP)	[^3^H](+)PN 200-110	0.04	nifedipine/1	90/22	rat cerebral cortex
Ca^2+^channel (L,diltiazem)	[^3^H]diltiazem	5	diltiazem/10	120/22	rat cerebral cortex
Ca^2+^channel (L,verapamil)	[^3^H] (–)D 888	3	D 600/10	120/22	rat cerebral cortex
K+ATP channel	[^3^H]glibenclamide	0.1	glibenclamide/1	60/22	rat cerebral cortex
K+V channel	[^125^I]α−dendrotoxin	0.01	α−dendrotoxin/50	60/22	rat cerebral cortex
SK+Cachannel	[^125^I]apamin	0.007	apamin/0.1	60/4	rat cerebral cortex
Na+channel (site 2)	[^3^H]batrachotoxinin	10	veratridine/300	60/22	rat cerebral cortex
Cl- channel (GABA-gated)	[^35^S]TBPS	3	picrotoxinin/20	120/22	rat cerebral cortex
NE transporter *(h)*	[^3^H]nisoxetine	1	desipramine/1	120/4	CHO cells
DA transporter *(h)*	[^3^H]BTCP	4	BTCP/10	120/4	CHO cells
GABA transporter	[^3^H]GABA	10	GABA/1	30/22	rat cerebral cortex
Choline transporter *(h)*	[^3^H]hemicholinium-3	3	hemicholinium-3/10	60/22	CHO cells
5-HT transporter *(h)*	[^3^H]imipramine	2	imipramine/10	60/22	CHO cells

Analysis and expression of results are described in Materials and Methods. Conc. – concentration; Inc. – incubation.

In the current study, we further investigated the molecular mechanism of action and signaling pathways of TVP1022 which may account for the cyto/cardio-protective efficacy of this drug. Here, using specific receptor binding and enzyme assays, our findings demonstrated that imidazoline 1 and 2 binding sites (I_1_ & I_2_) are potential targets for TVP1022. Focusing on the role of the I_1_imdazoline receptor in the mechanism of action of TVP1022, we deciphered the intracellular effect of the drug on the MAPK signaling pathway coupled to I_1_imidazoline receptor in rat pheochromocytoma PC12 cells and cultured NRVM. Our findings suggest that the I_1_imidazoline receptor represents a novel site of action for the cyto/cardio-protective efficacy of TVP1022.

**Table 2:** Summary binding assays results for TVP1022.

Cerep Compound I.D	% Inhibition of Control Specific Binding
A_1_ *(h)*	−13
A_2A_ *(h)*	10
A_3_ *(h)*	−37
α_1_ (non-selective)	5
α_2_ (non-selective)	32
β_1_ *(h)*	2
β_2_ *(h)*	−10
AT_1_ *(h)*	−19
AT_2_ *(h)*	−8
BZD (central)	6
B_1_ *(h)*	9
B_2_ *(h)*	−1
CB_1_ *(h)*	−15
CB_2_ *(h)*	4
CCK_A_ *(h)* (CCK_1_)	−18
CCK_B_ *(h)* (CCK_2_)	−3
CRF_1_ *(h)*	−37
D_1_ *(h)*	9
D_2S_ *(h)*	2
D_3_ *(h)*	3
D_4.4_ *(h)*	4
ET_A_ *(h)*	−107
ET_B_ *(h)*	−3
GABA (non-selective)	3
AMPA	15
Kainate	1
NMDA	3
H_1_ *(h)*	2
H_2_ *(h)*	1
H_3_ *(h)*	−1
I_1_	**89**
I_2_	**80**
LTB_4_ *(h)* (BLT_1_)	4
LTD_4_ *(h)* (CysLT_1_)	−9
MC_4_ *(h)*	5
M (non-selective)	3
NK_1_ *(h)*	2
NK_2_ *(h)*	−5
NK_3_ *(h)*	−2
Y (non-selective)	2
N(neuronal)(α-BGTX insensitive) (α4β2)	1
Opioid (non-selective)	17
ORL1 *(h)* (NOP)	21
PPARγ*(h)*	−34
PCP	−6
EP_4_ *(h)*	1
IP *(h)* (PGI_2_)	−36
P2X	−2
P2Y	−1
5-HT (non-selective)	**72**
σ (non-selective)	50
Glucocorticoid *(h)* (GR)	1
Estrogen *(h)* (ER)	−7
Progesterone *(h)* (PR)	−10
Androgen *(h)* (AR)	1
TRH_1_ *(h)*	−8
V_1a_ *(h)*	8
V_2_ *(h)*	1
Ca^2+^channel (L, DHP site)	19
Ca^2+^channel (L, diltiazem side) (benzothiazepines)	7
Ca^2+^channel (L,verapamil site) (phenylalkylamines)	3
K+ATP channel	19
K+V channel	−6
SK+Cachannel	13
Na+channel (site 2)	24
Cl- channel (GABA-gated)	9
NE transporter *(h)*	9
DA transporter *(h)*	8
GABA transporter	−11
Choline transporter *(h)* (CHT1)	19
5-HT transporter *(h)*	6

The binding assays results for TVP1022 (10 µM) are expressed as a percent of control specific binding [(measured specific binding/control specific binding) × 100] and as a percent inhibition of control specific binding [100−((measured specific binding/control specific binding) × 100)] obtained in the presence of TVP1022, as described in Materials and Methods.

## Materials and Methods

### Materials

TVP1022 was kindly donated by TEVA (Netanya, Israel). Efaroxan (a selective I_1_ imidazoline receptor antagonist), moxonidine (an I_1_ imidazoline receptor agonist) and β-actin antibody were purchased from Sigma Chemical Co. (St. Louis, MO, USA). Anti-phospho-p42/44 mitogen-activated protein kinase (MAPK), anti-p42/44 MAPK and cleaved caspase 3 antibodies were purchased from Cell Signaling Technology (Beverly, MA, USA). The specific inhibitor of MAPK activation, PD98059 was obtained from Calbiochem (La Jolla, CA, USA). Tissue culture reagents were obtained from Beth-Haemek Industries (BeitHaemek, Israel).

**Figure 1:**
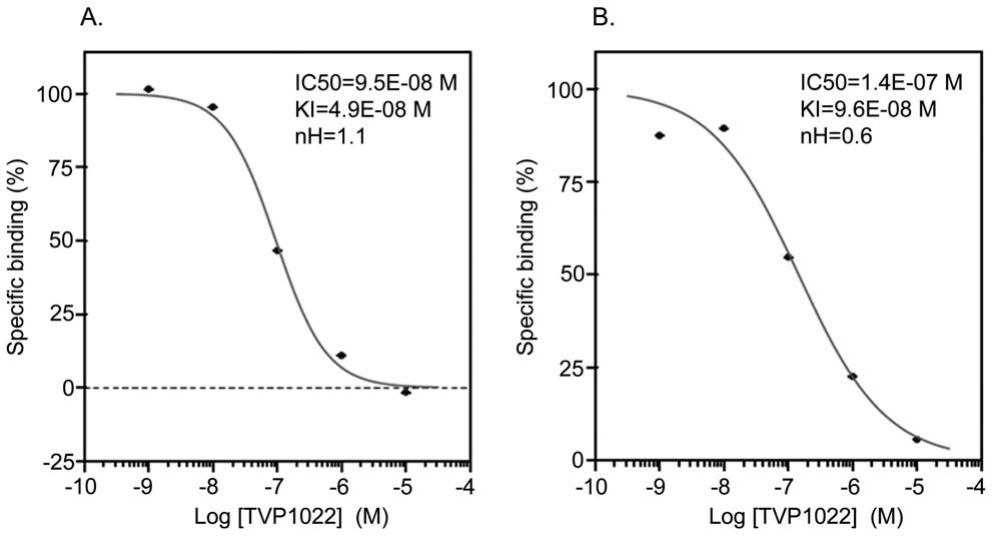
Competition curves obtained with TVP1022 at the I_1_ and I_2_imidazoline receptors. A. I_1_ imidazoline receptor and B. I_2_ imidazoline receptor competition curves demonstrating the IC_50_ values were determined by non-linear regression analysis generated with mean replicate values. For the determination of respective I_1_ and I_2_ imidazoline receptor binding assays: bovine adrenal medulla glands and rat cerebral cortex were incubated with [^3^H]clonidine (15 nM) [28] and [^3^H]idazoxan (2 nM) [29] in the absence or presence of TVP1022 (10E^−9^ M - 10E^−5^ M) for 60 min in 22°C or 30 min in 22°C, respectively. The specific ligand binding to the receptors is defined as the difference between the total binding and the nonspecific binding determined in the presence of an excess of unlabelled ligand, as described in Table 1.

**Table 3:** The binding values of I_1_ and I_2_ imidazoline receptors of reference compounds.

*n*H	K_i_(M)	IC_50_(M)	Assay/Reference compound
0.9	8.4E-08	1.6E-07	I_1_/rilmenidine
1.7	1.0E-08	1.6E-08	I_2_/idazoxan

The IC_50_,K_i_ and *nH*values were calculated as described in Materials and Methods.

### In vitro Pharmacology: a Diversity Profile Study of TVP1022

A broad spectrum receptor binding analysis was carried out on isolated receptors and binding sites. The specific ligand binding to the receptors is defined as the difference between the total binding and the nonspecific binding determined in the presence of an excess of unlabelled ligand (Table 1). The IC_50_ values (concentration causing a half-maximal inhibition of control specific binding) and Hill coefficients (*nH*) were determined by non-linear regression analysis of the competition curves generated with mean replicate values using Hill equation curve fitting [Y = D+((A – D)/(1+(C/C50) nH)], where Y = specific binding, D = minimum specific binding, A = maximum specific binding, C = compound concentration, C50 = IC_50_, and nH = slope factor). This analysis was performed using SigmaPlot ® 4.0 for Windows ® (© 1997 by SPSS Inc.). The inhibition constants (K_i_) were calculated using the Cheng Prusoffequation [K_i_ = IC_50_/(1+(L/K_D_)], where L = concentration of radioligand in the assay, and K_D_ = affinity of the radioligand for the receptor). Reference compounds: In each experiment, the respective reference compound was tested concurrently with TVP1022 in order to assess the assay suitability and was tested at several concentrations (for EC_50_ or IC_50_ value determination), and the data were compared with historical values determined at Cerep (Poitiers, France Laboratories, Le bois l'Evêque 86600 Celle l'Evescault, France) (Table 1). The assay was rendered valid if the results of the reference compounds fell within the specifications as defined in the corresponding standard operating procedure.

**Figure 2:**
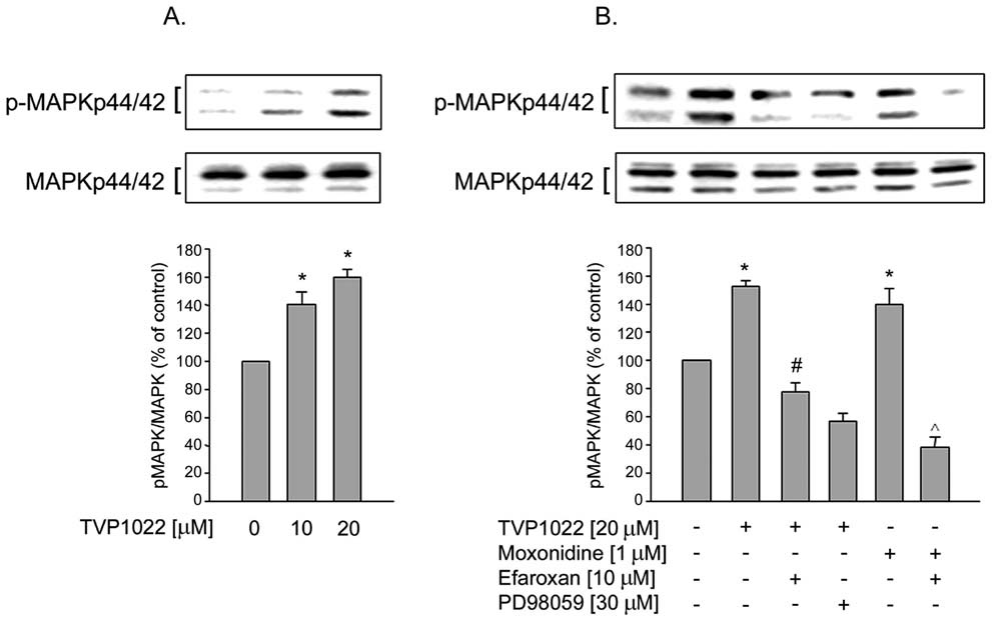
The effect of TVP1022 on MAPK activation in PC12 cells. A. PC12 cells were treated without or with TVP1022 (10 and 20 µM) for 30 min. B. PC12 cells were pre-incubated for 30 min with vehicle alone, or with efaroxan (10 µM) or with PD98059 (30 µM ) and then incubated without or with TVP1022 (20 µM) or moxonidine (1 µM ) for 30 min. Phosphorylation of MAPK was analyzed in cell lysates and the loading of the lanes normalized to levels of non-phospho MAPK. Results are expressed as mean±SEM (n = 3–4). *p<0.05 *vs.* control; #p<0.05 *vs.* TVP1022 alone; ∧p<0.05 *vs*. moxonidine alone.

### Cell Cultures and Viability Assay

PC12 cells originated from rat pheochromocytoma purchased from ATCC, were grown to confluence in T75 flasks, containing DMEM (1000 mg/l glucose) and supplemented with 5% fetal calf serum (FCS), 10% horse serum, and a mixture of 1% of penicillin/streptomycin/nystatin [11]. NRVM cultures were prepared from ventricles of 1–2 day old Sprague-Dawley rats, as previously described (ethics number Il-106-11-2007) [12]. Briefly, the ventricles of the excised hearts were dissociated with 0.1% RDB, a protease isolated from fig tree extract (Institute of Biology, Nes-Ziona,Israel), and the dispersed cells were resuspended in F-10 culture medium containing 1 mM CaCl_2_, 100 U/mL penicillin-streptomycin, 5% FCS, 5% donor horse serum, and 25 mg of bromodeoxyuridine (5-bromo-2′-deoxyuridine, BrdU).Cell cultures were incubated at 37°C in a humid 5% CO_2_-95% air environment. For all experiments, cells were initially seeded in full-serum cultured media and then replaced into serum-free media containing the respective supplements.

**Figure 3:**
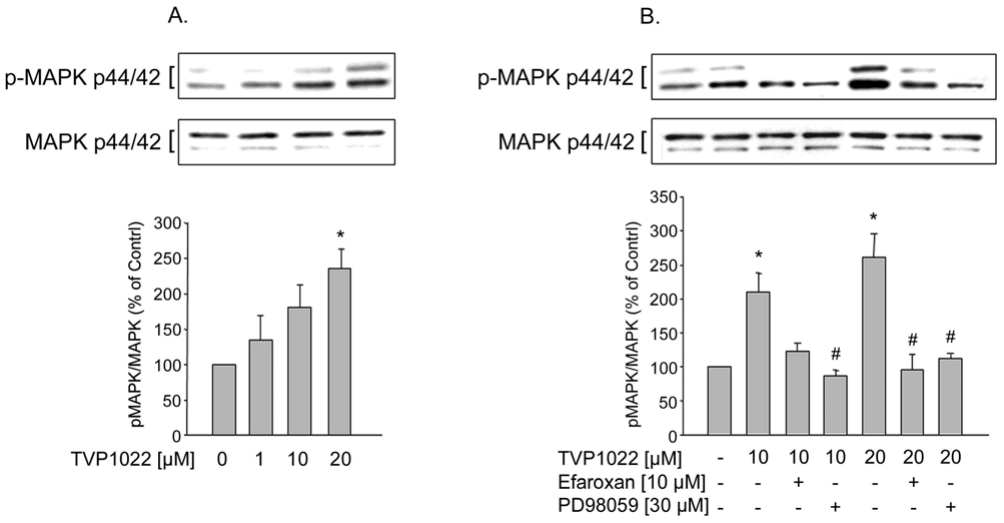
Effect of TVP1022 on MAPK activation in NRVM. A. NRVM cells were incubated for 30 min without or with increasing concentrations of TVP1022. B. NRVM cells were pre-incubated for 30 min with vehicle alone, or with PD98059 (30 µM) or efaroxan (10 µM) and then incubated without or with TVP1022 10 µM or 20 µM for 30 min. Phosphorylation of MAPK was analyzed in cell lysates and the loading of the lanes normalized to levels of non-phospho MAPK. Results are mean±SEM values of (n = 4) independent experiments. *p<0.05 *vs.* control, #p<0.05 *vs.* TVP1022.

*In situ* DNA fragmentation was performed by the terminal deoxynucleotidyl transferase-mediated deoxyuridine triphosphate-digoxigenin nick end labeling (TUNEL). NRVM were incubated without or with TVP1022 (20 µM) in serum free medium for 24 h. Cells were fixed in 4% paraformaldehyde, permeabilized with Triton X-100 (0.1%) and then stained with TUNEL kit (Roche, Penzberg, Germany) following by 4′-6-Diamidino-2-phenylindole (DAPI). Confocal image stacks were captured with a Zeiss LSM-5, Axiovert 200 microscope, using LSM 5 analysis software (Zeiss, Oberkochen, Germany). Each test group was performed at least four times in 70–100 cells. Quantification of the apoptotic cells was calculated as the ratio of TUNEL-positive cells to the total number of cells in the same slide.

**Figure 4:**
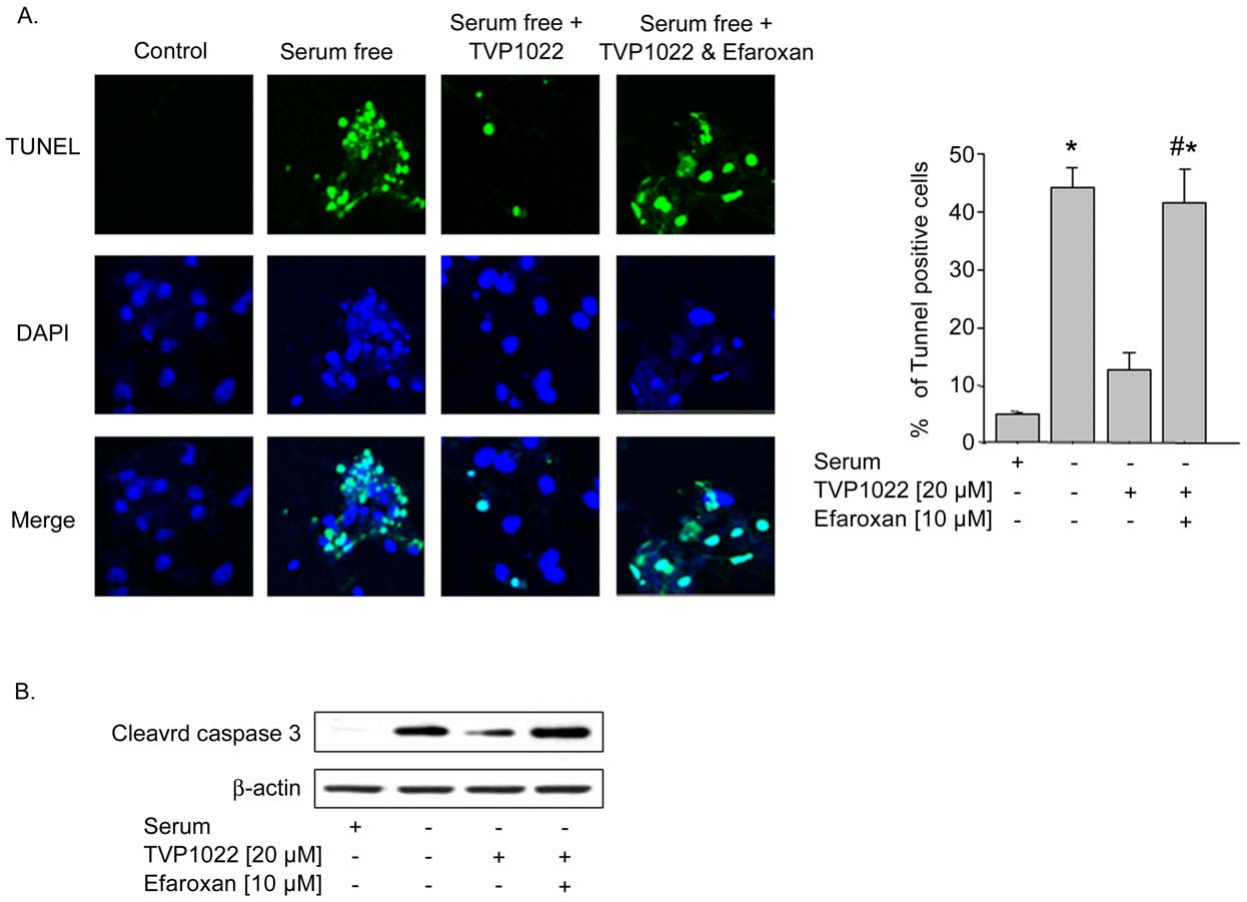
Cardioprotective effect of TVP1022 in NRVM. NRVM cells were incubated in full serum medium for 24 h before replacing to serum free medium. Subsequently, the cells were incubated with vehicle alone, or with efaroxan (10 µM), followed by incubation without or with TVP1022 (20 µM) for additional 24 h. A. Apoptotic nuclei were identified by TUNEL analysis. Absolute values of 5–10 separate fields were averaged, and apoptotic cells were expressed as percentage of total cells in 3 independent experiments. *p<0.05 *vs.* full serum; #p<0.05 *vs*. serum free+TVP1022. B. Representative Western blots and quantitative results of cleaved caspase 3 protein expression. The loading of the lanes was normalized to β-actin levels.

### Western Blot Analysis

PC12 cells or NRVM cultures were resuspended in RIPA (20 mM Tris–HCl pH 7.4, 200 mM NaCl, 1% Triton-X 100, 0.1% SDS, 0.2% sodium deoxycholate, 5 mM EDTA) containing cocktail protease inhibitors (Roche, Basel Switzerland). The lysates were precleared by centrifugation. Protein content was determined using the Bradford method (Sigma Chemical Co. St. Louis, MO, USA).Equal amounts of sample were resolved on sodium dodecyl sulfate-polyacrylamide gel electrophoresis (SDS-PAGE) and blotted onto polyvinylidenedifluoride membranes (Millipore, Billerica, MA, USA). Membranes were treated with blocking buffer (5% dry milk, 0.05% Tween 20 in TBS). Primary antibodies were diluted in the blocking buffer and incubated with membranes for 20 h at 4°C followed by incubation (1 h at room temperature) in dilutions of horseradish peroxidase-conjugated secondary antibodies in the same buffer. Following antibody incubations, membranes were washed in 0.5% Tween 20 TBS. Detection was achieved using Western blotting ECL reagent (Amersham, Pharmacia, Little Chalfort Buckinghamshire, UK). Quantification of the results was accomplished by measuring the optical density of the labeled bands from the autoradiograms, using the computerized imaging program Bio-1D (VilberLourmat Biotech. Bioprof., France). The values were normalized to β-actin intensity levels.

### Statistical Analysis

Each experiment was repeated 3–4 times in triplicates and data are expressed as mean±SEM. For statistical analysis one-way analysis of variance followed by Student's *t* test was performed and a value of p<0.05 was considered significant.

## Results

### The Binding Properties of TVP1022

The binding sites characterization of TVP1022 was accomplished by receptor binding and enzyme assays, as described in the Materials and Methods section. In each experiment, the respective reference compound was tested concurrently with TVP1022 in order to assess the assay suitability. TVP1022 was tested at several concentrations (1E-09-1E-05 M; for EC_50_ or IC_50_ value determination), and the data regarding the binding values of I_1_ and I_2_ imidazoline receptors were summarized and compared with historical values determined at Cerep (see Table 2). At 10 µM TVP1022, results showing an inhibition (or stimulation for assays run in basal conditions) higher than 50% are considered to represent significant effects of the test compounds. Fifty percent is the most common cutoff value for further investigation (determination of IC_50_ or EC_50_ values from concentration response curves). Results showing an inhibition (or stimulation) between 20% and 50% are indicative of weak to moderate effects (in some assays, they may be confirmed by further testing as they are within a range where more inter-experimental variability can occur). Results showing an inhibition (or stimulation) lower than 20% are not considered significant, and are mostly attributable to variability of the signal around the control level. Low to moderate negative values have no real meaning and are attributable to variability of the signal around the control level.

For TVP1022 (10 µM), the percent inhibition of control specific binding was found to be 89% and 80% for I_1_ and I_2_ imidazoline receptors, respectively, and 72% for 5-HT (Table 2). The values of IC_50_, K_i_ and *n*H determined for TVP1022 binding to I_1_ and I_2_ are shown in Figure 1. These binding values were also indicated and compared for each reference compound (Table 3), and the results were within the accepted limit of historic average ±0.5 log units.

### The Effect of TVP1022 on p42/44 MAPK Activation in Extracts from PC12 Cells

Previous studies showed that MAPK activation is pivotal to the protective activity of various propargyl derivatives [6], [15]. Here, we further investigated the effect of TVP1022 on MAPK signaling pathway known to be coupled to I_1_ imidazoline receptor in PC12 cells [16], [17], [18]. This cell line provides the predominant cellular model for investigating I_1_ imidazoline receptor signaling [16]. As shown in Figure 2A, TVP1022 (10 and 20 µM) dose-dependently increased the immunoreactivity of the phosphorylated p42 and p44 MAPK in PC12 cells (at 20 µM, 152.2±4%, n = 3, p<0.05, compared to control levels), whereas the total amount of MAPK was constant.

Next, we investigated the inhibitory potential of PD98059 (a non-competitive inhibitor of MEK1 phosphorylation and activation) on TVP1022-induced MAPK phosphorylation. As shown in Figure 2B, pretreatment with PD98059 (30 µM) significantly blocked the effect of TVP1022-induced increase of MAPK phosphorylation.The activation of p42/44 MAPK was determined as the ratio of the amount of the dually phosphorylated active form to total MAPK immunoreactivity. In addition, to analyze whether the effect of TVP1022 on MAPK stimulation was mediated by the I_1_ imidazoline receptor, we used efaroxan, a selective I_1_ imidazoline receptor antagonist [18]. As depicted in Figure 2B, efaroxan (10 µM) abolished the effect of TVP1022 on MAPK activation, suggesting the involvement of I_1_ imidazoline receptor in TVP1022-induced MAPK activation. Confirming these results [16], monoxidine (1 µM), an I_1_imidazoline receptor agonist, increased the phosphorylation of p42 and p44 MAPK, which was also abolished by efaroxan (Fig. 2B).

### TVP1022 Increased ERK Activation in NRVM via the I_1_ Imidazoline Receptor

Compatible with the results showing the involvement of the I_1_ imidazoline receptor in TVP1022-induced activation of MAPK in PC12 cells, we further tested whether TVP1022 can activate this signaling pathway in NRVM cultures. Indeed, the phosphorylation of both MAPK isoforms was markedly increased in NRVM treated with TVP1022 (at 20 µM, 236±27%, n = 3, p<0.05, compared to control levels) (Fig. 3A). Accordingly, PD98059 (30 µM) abolished TVP1022-induced ERK phosphorylation (Fig. 3B). At both 10 and 20 µM, TVP1022-mediated increases in p42 and p44 MAPK phosphorylation were antagonized by efaroxan (Fig. 3B). These findings clearly demonstrate that the I_1_imidazoline receptor is involved in TVP1022-mediated p42 and p44 MAPK activation in NRVM cultures.

### I_1_ Imidazoline Receptor Mediated TVP1022-induced Cytoprotection in NRVM

As TVP1022 was recently shown to protect NRVM against various oxidative stress-induced insults [12], [13], we tested whether the I_1_imidazoline receptor mediates the cytoprotective effect of TVP1022 in NRVM. The current cytoprotective studies complement our previous observations, showing that TVP1022 possesses the capacity to protect NRVM from serum deprivation-induced apoptosis [12]. In the present study, NRVM cultures were grown in serum-free medium in the absence or presence of TVP1022. As shown in Figure 4A, the pro-apoptotic effect of serum deprivation is reflected by the significant increase of apoptotic cells, compared to control, full serum cultures. In agreement with its cytoprotective efficacy, TVP1022 (20 µm) decreased the number of apoptotic cells, as was detected by TUNEL (Fig. 4A). Pre-incubation of NRVM with the efaroxan, markedly prevented TVP1022-induced cytoprotective effect (Fig. 4A). Consistent with its anti-apoptotic effect, TVP1022 attenuated the appearance of the cleaved, activated forms of caspase 3, whereas efaroxan markedly reversed TVP1022-suppressive effects on caspase 3 (Fig. 4B). Collectively, these data suggest that TVP1022 cytoprotection against serum deprivation is mediated through the I_1_ imidazoline receptor.

## Discussion and Conclusions

Our studies have previously shown that TVP1022, the S-enantiomer of rasagiline (Azilect®) (N-propargyl-1R-aminoindan) features cyto/cardio-protective efficacy in a variety of experimental models. Using specific receptor binding and enzyme assays, the present findings demonstrated that I_1_ andI_2_ imidazoline binding sites are potential targets for TVP1022 (IC_50_ = 9.5E-08 M and IC_50_ = 1.4E-07 M, respectively). Consequently, the results of the present study also showed that TVP1022 induced a significant increase in the phosphorylated MAPK p42/44 levels in rat pheochromocytoma PC12, which are the predominant cellular model for investigating the I_1_ imidazoline receptor signaling pathway [16], [19]. It was previously shown by radioligand binding and by molecular approaches that PC12 cells express I_1_ imidazoline receptors, but lack both α_2_-adrenergic and I_2_ imidazoline receptors [20], [21]. Previous experiments on PC12 cells suggested that the I_1_ imidazoline receptors belong to the sphingosine -1-phosphate (S1P)- receptor family, representing a mixture of S1P_1_/S1P_3_ [21], [22]. Indeed, it was shown that S1P, the endogenous ligand for these receptors, decreased the heart rate, ventricular contraction and blood pressure in the *in vivo* rat model [23].

Previous studies have established a link between the I_1_ imidazoline receptor and the MAPK signaling pathway. Thus, activation of the I_1_ imidazoline receptor, which is coupled to phosphatidylcholine selective phospholipase C (PC-PLC), was shown to result in down-stream activation of MAPK p42/44 [24]. It was reported that stimulation of the I_1_ imidazoline receptor in PC12 cultures with the agonist moxonidine lead to PC-PLC activation [20], [25], [26]. Additionally, activation of PC-PLC by imidazoline agonists resulted in increased formation of the second messenger diacylglycerol from phosphatidylcholine and the release of phosphatidylcholine. These effects can be blocked by efaroxan, which was shown to antagonize the PL-PLC pathway associated with I_1_ imidazoline receptor [24]. Indeed, in the current study, we found that in PC12 cells, the effect of TVP1022 was prevented by efaroxan, indicating the involvement of I_1_ imidazoline receptor in TVP1022-mediated p42/44 MAPK phosphorylation in these cells.

In this study, we also explored the potential effect of TVP1022 on MAPK signaling pathway coupled to I_1_imidazoline receptor in cultures of NRVM. The data clearly indicate that the drug specifically stimulated p42/44 MAPK phosphorylation in NRVM. Moreover, the ability of the imidazoline I_1_-receptor antagonist efaroxan to block TVP1022-induced MAPK phosphorylation in NRVM further suggests that this effect of the drug is a receptor-mediated effect. While PC12 cells are deficient in α_2_-adrenoceptors, which renders them a suitable model for investigatingI_1_ imidazoline receptor signaling [20], [21], the potential contribution of α_2_-adrenoceptors to TVP1022 effects in NRVM cannot be ruled out.

Importantly, an additional observation of the present study is that the cytoprotective effect of TVP1022 demonstrated in NRVM against serum deprivation-induced toxicity was markedly inhibited by efaroxan, thus suggesting the importance of the I_1_ imidazoline receptor in mediating the cytoprotective activity of the drug. Thus, in NRVM under cytotoxic, serum deprivation conditions, efaroxan blocked the protective effect of TVP1022 determined in terms of both the reduction of apoptotic cells and decrease of caspase 3 activity, as assessed by the levels of cleaved forms of caspase 3. Indeed, the cytoprotective potency of TVP1022 shown in NRVM is in agreement with our previous studies, demonstrating that TVP1022 attenuated serum deprivation-induced apoptosis, including inhibition of the increase in cleaved caspase3 levels and prevention of the decline in Bcl_2_/Bax ratio [12].

Interestingly, it was recently reported that in addition to its sympathetic inhibition, the I_1_ imidazoline receptor agonist, monoxidine may also directly regulate molecular and functional processes in the heart, including anti-apoptotic cardiac effects and improvement of cardiac performance [27]. In summary, our present findings indicate that I_1_ imidazoline receptors area potential molecular target for TVP1022.It is suggested that I_1_ imidazoline receptors are involved in the cytoprotective beneficiary activity of TVP1022,further clarifying the mechanism of action associated with the cardioprotective effect of TVP1022.
