# Correction: I_1_ Imidazoline Receptor: Novel Potential Cytoprotective Target of TVP1022, the S-Enantiomer of Rasagiline

**DOI:** 10.1371/annotation/9dae97ef-4a68-47b2-a8f5-fb953ebe877c

**Published:** 2013-06-10

**Authors:** Yaron D. Barac, Orit Bar-Am, Esti Liani, Tamar Amit, Luba Frolov, Elena Ovcharenko, Itzchak Angel, Moussa B. H. Youdim, Ofer Binah

There was an error the column arrangement of Table 3. Please see the corrected table at the following link:

**Figure:**
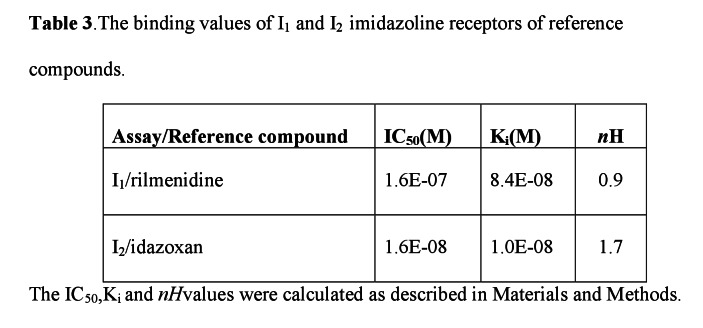


.

